# Giant hepatic hydatid cyst with sub-fascial extension treated by open minimally invasive surgery: a case report

**DOI:** 10.1186/1752-1947-2-26

**Published:** 2008-01-28

**Authors:** Dipesh D Duttaroy, Samir Kacheriwala, Bithika Duttaroy, Jitendra Jagtap, Gunjan Patel, Nikhil Modi

**Affiliations:** 1 Department of Surgery, Government Medical College & Sir Sayajirao General Hospital, Baroda, Gujarat, 390001, India; 2 Department of Microbiology, Government Medical College & Sir Sayajirao General Hospital, Baroda, Gujarat, 390001, India

## Abstract

**Introduction:**

Hepatic hydatid disease can be successfully treated by a variety of modalities.

**Case Presentation:**

We report a case of a 60 year old male with giant hepatic hydatid disease who presented with a huge cystic mass in the upper abdomen. Diagnosis was confirmed by serology, ultrasonography and CT scan. The patient was treated successfully by open minimally invasive surgery with minimum breaching of the peritoneal cavity using a laparoscopic trocar to evacuate the cyst.

**Conclusion:**

The use of a laparoscopic trocar through a small abdominal incision in selected patients with hepatic hydatid disease with subfascial extension can be a safe, minimally-invasive option of treatment

## Introduction

Cystic hydatid disease (echinococcosis) is an important zoonotic disease caused in humans by Echinococcus granulosus, a cestode that usually inhabits the intestine of dogs and other canines as a definitive host. Humans are accidental intermediate hosts due to ingestion of the parasitic eggs. The liver is the most common site for the occurrence of the larval form of cystic hydatid disease, the others being lung, brain and other viscera [1]. Though a variety of treatment modalities have been successfully employed, there is a lack consensus as to the most appropriate method. Medical therapy in the form of benzoimidazole carbamates alone or in combination with praziquantel has been advocated for the treatment of hydatid disease [2-4]. Interventional radiologists and gastroenterologists have used minimal invasive procedures such as PAIR (puncture, aspiration, injection, re-aspiration) [5-7] and PEVAC (percutaneous evacuation of cyst content) [8] for treating hepatic echinococcosis. An array of surgical procedures has been recommended. In recent times, laparoscopic surgery and the use of laparoscopic instruments (trocar and suction) have been found to be safe and effective in the management of hepatic hydatid disease [9-11]. We report a patient with giant hepatic hydatid disease with subfascial extension into the abdominal wall who was treated successfully by open minimal invasive surgery with minimum violation of the peritoneal cavity.

## Case presentation

**Figure 1:**
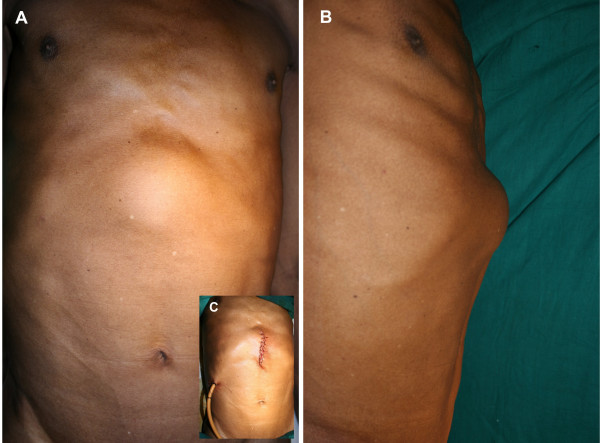
A) Anterior view of abdomen showing the globular cystic lump in the epigastrium. B) Left lateral view abdomen showing the lateral profile of the lump. C) Inset: Sutured incision with Foleys catheter in situ, draining the cyst cavity.

**Figure 2:**
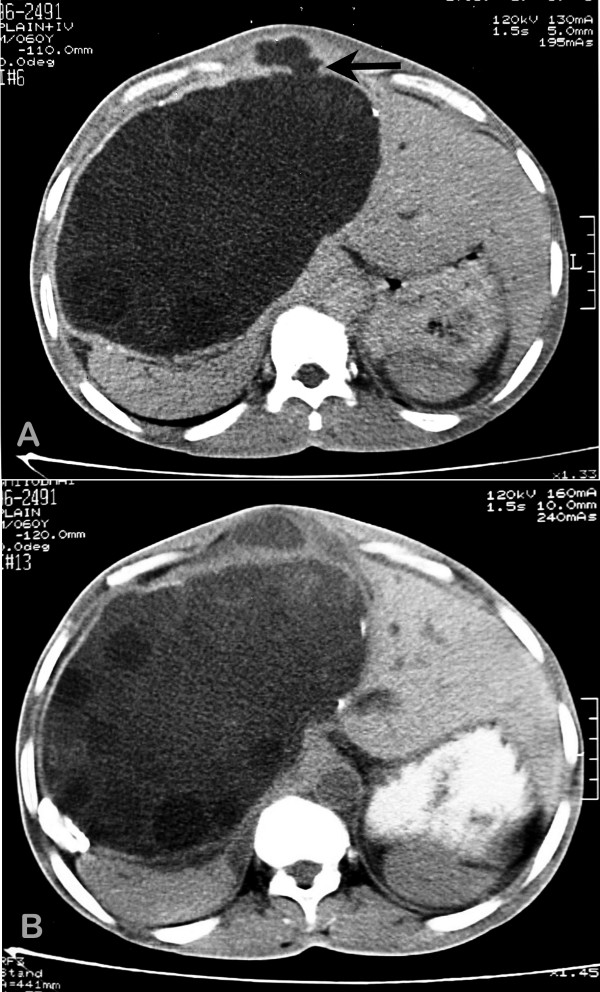
A) & B) Axial spiral CT scan of abdomen through two different levels showing a hydatid cyst in the left lobe (Segments V, VI, VII, VIII) with a cystic projection anteriorly.

A 60-year-old male presented with continuous dull aching upper abdominal pain of four months duration and a gradually increasing visible upper abdominal lump over the past two months. Clinical examination revealed an afebrile non-icteric man with mild pallor and pedal edema. The patient had a huge lobulated liver (span 22 cm) occupying both hypochondria and the right lumbar, epigastrium and umbilical regions with a localized cystic subfascial projection in the epigastrium of 8 × 8 cm. (Figure-1A &1B) Laboratory investigations revealed haemoglobin 10 gm%, white blood cell count 7500/μl and eosinophils 900/μl. Serological test with an enzyme-linked immunosorbent assay (ELISA) for echinococcus was positive. Liver function tests were within normal range. Radiography showed elevation of the right diaphragm with a soft tissue shadow in the upper abdomen. Ultrasonography (USG) of the abdomen revealed a 19 × 12 × 13 cm cystic lesion in the right lobe of the liver with multiple anechoic cysts within it. Spiral CT scan of the liver (Figure-2A &2B) confirmed a hydatid cyst in the right lobe (segments – V, VI, VII, VIII), with multiple daughter cysts within, compressing the portal vein, inferior vena cava, hepatic veins, gallbladder, intra and extra hepatic biliary tree and the right kidney. The anterior aspect of the cyst demonstrated a cystic projection in the midline stretching the fascial aponeurotic layer. (Black arrow – Figure-2A)

**Figure 3:**
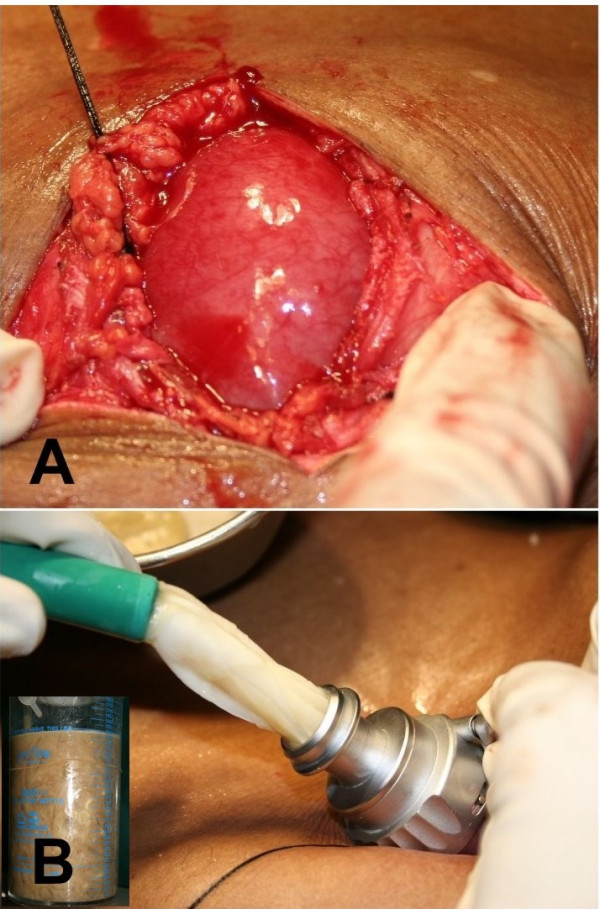
A) Intraoperative view showing the exposed cystic projection of the hydatid cyst with a purse string all around. B) Daughter cysts being sucked through the laparoscopic trocar sheath. (Inset: suction bottle containing cyst contents.)

The patient received Albendazole 10 mg/kg/day for 28 days with the aim of sterilizing the cyst contents. His abdomen was then explored under general anaesthesia using a 6 cm midline incision over the epigastric cystic swelling. After dividing the linea alba a close continuous suture of 2-0 silk was taken all around between the divided fascial aponeurosis and the projecting cyst wall (Figure-3A) to prevent the spillage and entry of cyst contents into the peritoneal cavity during the process of evacuation. Using a 16-gauge needle, a three-way stopcock, and a 50 ml syringe, we attempted aspiration of the cyst prior to instillation of a scolicidal agent through the exposed cyst wall. Due to the thick contents of the cyst, the attempt failed and was abandoned. We introduced a 10 mm laparoscopic trocar into the cyst cavity after stabilizing the exposed cyst wall with tissue forceps and isolating the area with gauze packs soaked in 0.5% Cetrimide solution. A suction cannula was applied to the mouth of the laparoscopic sleeve keeping the valve open. Alternate use of the suction tube applied to the mouth of the sleeve (Figure-3B) and a high pressure laparoscopic suction irrigation apparatus introduced into the cyst cavity resulted in a drainage of three liters of thick, viscid, cream-colored cyst contents containing abundant daughter cysts (Figure-3B inset). After near total evacuation of the cyst, a 30° telescope was introduced through the trocar sleeve to visualize the cavity for adherent membranes and biliary leak. Adherent daughter cysts and membranes were then evacuated manually by a long thin spoon introduced through the trocar site after its removal.

We could not visualize any gross biliary leak. The cyst cavity was irrigated with 2.5 liters of 1% povidone iodine solution twice. A 24 F self retaining Foleys catheter was then introduced into the cyst cavity as a drain, the course of which was routed with the help of a long Robert forceps passed though the opening into the cyst cavity and then rail-roaded into position. (Figure-1C inset) The trocar site on the cyst was closed by continuous 2-0 Polyglactin sutures. After local irrigation of the site with 0.5% Cetrimide solution, the silk suture anchoring the cyst wall to the fascial layer was then detached and the linea alba was closed with continuous 1-0 Polypropylene sutures. Patient received liquids orally in the evening and solid food next morning onwards. The patient had received an injection of cefotaxime perioperatively. He was discharged on the third postoperative day with the drain in situ.

The cyst contents, which were sterile on culture, showed protoscolices, fragments of laminated membrane and hooklets. Skin sutures were removed on the eighth postoperative day. Other than postoperative biliary drainage of 500 ml/day that gradually decreased over one month, the patient's recovery was uneventful. We removed the drain after USG confirmed a collapsed cyst cavity with minimal collection. Follow-up USG after a period of four months revealed a collapsed residual cavity with no evidence of recurrent disease. Though a CT scan is essential to compare the pre and postoperative characteristics in hepatic hydatid disease, it was not feasible due to financial constraints.

## Discussion

Despite a variety of open and minimal invasive techniques being available for the treatment of hydatid disease of the liver, one of the main concerns of the treating physician is spillage of cyst contents that can lead to recurrence in various forms and anaphylactic reactions. We cannot apply a single procedure uniformly because hepatic hydatid disease presents in diverse forms, which necessitates appropriate measures for each case. PAIR with benzoimidazole carbamates is recommended as a primary line of therapy for uncomplicated hepatic echinococcosis [5-7,12]. Though, there have been reports of successful percutaneous drainage of giant hepatic hydatid cyst, huge complicated cysts with impending rupture or fistulization are poor candidates for such intervention. In such instances, appropriate surgical management becomes vital.

Open surgical techniques employed include evacuation and simple closure, evacuation with drainage, marsupialization, closed total cystectomy, partial pericystectomy, partial pericystectomy with capitonnage, partial pericystectomy with cavity management (omentoplasty and internal drainage) and partial hepatectomy [11,12]. The principle of any surgical procedure for liver hydatid disease is complete evacuation of the cyst, prevention of intra-abdominal spillage, detection of major cysto-biliary communications, and sterilization and early obliteration of the residual cavity [11-13]. However, surgical procedures are not without complications and are associated with both morbidity (anaphylaxis, cyst infection, liver or intra-abdominal sepsis, haemorrhage and biliary fistula) and rarely mortality [7]. Over the last decade, laparoscopic management of liver hydatid disease has been carried out the world over with excellent results [9,11,12,14]. While, laparoscopic surgery follows all the principles of open surgery it is beneficial to the patient in providing reduced postoperative discomfort, shorter recovery time and reduced hospital stay.

In this case we approached the cyst directly since the anterior portion of the huge cyst was herniating into the midline as a diverticulum (Figure-2A Black arrow) and stretching the linea alba. Apprehensions about the spillage of the cyst contents into the peritoneal cavity prevented us from penetrating the cyst directly through the abdominal wall with a laparoscopic trocar. Our experience with advanced laparoscopic surgery is limited. Specialized instruments such as the Palanivelu Hydatid System [11] or the locking umbrella trocar, [14] which have been designed to prevent the spillage of hydatid fluid during laparoscopic surgery, were not available; hence, we avoided the conventional laparoscopic route. The open surgical technique adopted by us in this case offered most of the advantages of laparoscopic surgery. We could evacuate a giant hepatic hydatid cyst without intraperitoneal spillage, visualize the cavity and drain it through a small abdominal incision. Postoperative recovery time and hospital stay was reduced. The percutaneous laparoscopic approach has been adopted by Kayalp et al to deal with a liver abscess pointing onto the anterior abdominal wall in which the trocar was directly introduced into the abscess cavity [15]. The same author has used a laparoscopy trocar for evacuation of a hydatid cyst after conventional abdominal exploration through an extended subcostal incision with the aim of preventing spillage [10]. Seven et al have used the laparoscopic approach to enter the cyst cavity with a 10 mm trocar having an umbrella locking mechanism, that was utilized to suspend and fix the cyst against the abdominal wall [14]. This was subsequently followed by aspirating the cyst contents through the trocar, direct visualization of the cyst by introducing a telescope and drainage of the cyst. The advantage of their approach was that a biliary communication could be dealt with by laying open the cyst wall, which was not possible with our technique.

The advantage of our technique is that gross intra-peritoneal contamination is eliminated since the cyst is not exposed to the peritoneal cavity during surgery. The cyst contents, including daughter cysts, can be evacuated by high-pressure suction. If adherent membranes are visualized on the wall, they can be manually debrided through the same opening. The cyst is accessed through a small abdominal incision and there is no handling of abdominal viscera other than the liver. Postoperative pain and ileus is minimal leading to an early recovery. The patient can be started on oral fluids by the evening of surgery. One of the drawbacks is the potential risk of puncture of the cyst wall while taking the circumferential anchoring sutures between the fascia and the cyst wall leading to leak of hydatid fluid. Another limitation of the technique is that if cysto-biliary communications are visualized they cannot be dealt with intraoperatively without modifying the procedure. Postoperative biliary leakage has to be dealt with conservatively on expectant lines as in our patient, or by further interventional procedures. The introduction of the drain, though guided, is a blind procedure and can lead to potential injuries to the adjacent organs; hence utmost care has to be taken during its introduction. Ultrasound guided drain insertion may be a sound option if available. Though this method has been tried successfully in only a single patient we would like to emphasize that the same can be replicated in a selected subset of patients with large superficial palpable hydatid cysts either stretching or herniating through the abdominal wall musculature.

## Conclusion

The use of a laparoscopic trocar through a small abdominal incision in selected patients with hepatic hydatid disease can be a safe, minimally-invasive surgical option of treatment, which would reduce post operative discomfort and result in early recovery.

## Competing interests

The author(s) declare that they have no competing interests.

## Authors' contributions

DDD is the consultant surgeon responsible for the patient's care. He conceived this report, drafted the article and performed the surgery. SK assisted in performing the surgery, and helped in drafting and revision of the article. BD performed the investigations, helped in the literature search and supervised the drafting and overall structure of the article. JJ did the photography, helped in acquisition of data and technical support and revision of the article. GP acquired the radiological images and helped in drafting. NM performed the literature search and helped in revision. All authors read, appraised and approved the final manuscript.

## Consent

Written informed consent was obtained from the patient prior to publication of this case report.
